# Retrospect of the Medical Sciences

**Published:** 1843-10-14

**Authors:** 


### A NEW OPERATION FOR ARTIFICIAL PUPIL.

BY J. B. ESTLIN.

It is done with a small knife, used, I think, originally by Sir W. Adams, called an iris knife, and figured in the works of Mackenzie and others. By these authors it was used by being introduced behind the iris through the sclerotica, and then pushed through the iris into the anterior chamber.

I have been accustomed to use this little instrument differently, inserting it through the cornea, near the temporal canthus, close to the sclerotica, with the flat part of the blade towards the iris. The instrument being passed through the anterior chamber to its nasal extremity, the handle of the knife is turned a quarter of a circle, so as to bring its cutting edge against the iris, and it is then withdrawn by a quick movement, that depresses the point of the instrument upon the iris, so as to make a horizontal cut across that membrane.

I have found this mode of making an artificial pupil particularly successful in cases where the natural pupil has closed after extraction of the cataract. I have operated upon many persons so circumstanced, and have in an instant relieved all the disappointment of the patient and myself, at the apparent failure of an operation, in the result of which both were much interested. Of course a circular and handsome pupil cannot be ensured by this proceeding, though such will often be the result; but I am inclined to believe that those surgeons whose engagements have led them to deal much with cases of closed pupil, will be very thankful, as I am, to see a good hole in the iris, whatever its shape or size may be. I have seldom known any inflammation result from this method of dividing the fibres of the iris.

To one class of cases of fixed and nearly closed pupil, with cataract, this operation is peculiarly appropriate; indeed, more so than any other I am acquainted with. I have succeeded in giving very useful vision in such instances, where little hope of success had been entertained by other surgeons; or myself. I refer to those patients in whom chronic inflammation of the iris has accompanied the formation of cataract, where only a little pupillary aperture is left, and that of an irregular shape, the iris being apparently thinned, and its posterior surface glued down to the opaque crystalline capsule, often with portions of detached pigment tinging its surface, and the eye possessing so low a perception of light, as to excite much apprehension of the existence of amaurosis. (I would wish it to be clearly understood that in all the cases I am speaking of as fitted for operation, I presume that the patient is totally blind, for, while useful vision exists in one eye, I rarely advise any operation upon the other.)

In such hopeless cases, then, supposing all inflammation and headache long to have subsided, the operation I am describing is well worthy of trial. It is to be performed exactly as before detailed. The cataract behind is often solid, and affords a good resistance to the knife in cutting the fibres of the iris. It is uncertain what will be the effect of the sudden incision. I have sometimes by one cut divided the fibres of the iris, and displaced the cataract, so that a clear pupil was instantly produced, and a sudden blaze of light let in upon the retina, quite astounding to the delighted patient. At other times a permanent aperture in the iris will be made, of sufficient extent to allow of a thorough view of the opacities behind the pupil, and to admit of future operations with the needle for their removal, either through the cornea or sclerotica. I lay claim to no credit for this operation, having seen Mr. Alexander perform it thirty years ago; but I am not aware of its being described in anv works upon diseases of the eye, and, as I consider it a very valuable one, I am glad to avail myself the opportunity this meeting affords of making it more generally known.—*Prov. Med. Journ.*

---

### STATISTICS OF PUERPERAL CONVULSIONS.

I was for many years persuaded that puerperal convulsions only happened when the head presented, but experience has proved that they sometimes occur in preternatural presentations of the child. Of 19 cases recorded by Dr. Joseph Clarke, 16 were of first children; and such was the case in thirty-six instances out of forty-eight related by Dr. Merriman. Of 30 cases which occurred to Dr. Collins, 29 were in women with their first children, the other single case was in a second pregnancy, but in a woman who had suffered a similar attack in her first. Out of the 32 children—there being two twin cases—14 were born alive.—*Dr. R. Lee in Lon. Lancet.*

---

### NEW ASTRINGENT PRINCIPLE OF RHATANY.

By digesting rhatany root in sulphuric æther, a brown extract is obtained, which is perfectly soluble in distilled water, and causes a powerful sensation of astringency, followed by heat and dryness, when placed on the tongue. This extract, invented by M. Tissier, of Lyons, has been employed with success in that city in passive hæmorrhages, particularly those consequent on non-contraction of the uterus, occurring after prolonged labours and miscarriages. It has also been used with advantage for leucorrhœa, blenorrhœa, gleet, &c. The dose in which it has hitherto been employed, is a tablespoonful of a mixture composed of from five to ten grains of the extract in six ounces of some appropriate vehicle. In leucorrhœa, topical injections are recommended of from two to five grains of the extract in a pint of barley-water. The presence of this preparation in the stomach gives rise generally to a sensation of heat in the epigastrium, though this rarely proceeds so far as to become painful; great thirst, and a pulse often as full as in gastritis also prevail. These symptoms are, however, transient, and rapidly quelled by lemonade, or other mild drinks. Should the injection irritate the urethra too greatly, it is only necessary to suspend its use for a short time.—*Gaz. des Hopitaux.*

### RETROSPECT OF THE MEDICAL SCIENCES.

### MR. ESTLIN ON INJURIES TO THE IRIS.

Thirty years of experience in rather an extensive field of observation have strongly impressed upon my mind the remarkable power of the iris to bear injuries without destructive inflammation to that membrane, or to other textures of the eye. The highly vascular and nervous structure of the iris would lead to the expectation, *à priori*, that no injury could be inflicted upon it without giving rise to serious and disorganising inflammation, but I have seen almost every possible kind of violence done to it, with no more important consequences than such as the direct mechanical injury, produced at the moment of the accident, has occasioned. These accidents consist of lacerations of this membrane, cuts, punctures, separation from its attachments, together with protrusion through wounds of the cornea and sclerotica, and through openings occasioned by perforating ulcers of the cornea, in nearly all of which herniæ of the iris, the prolapsed portion becomes united to the wounded part.

As a type of the sort of injury the iris will meet with without becoming seriously inflamed, I will state the following:—

A young man, while turning iron, was struck on the right eye by a portion of the metal detached with violence from the lathe. The cornea was cut about the fifth of an inch in extent near the sclerotica, towards the outer canthus. The iris was lacerated, and some of it hung out of the wound. I removed the separated portion, which, when spread out, proved to be nearly half the iris. The patient walked to the dispensary, and continued to attend regularly as an outpatient. No inflammation supervened, the wound healed favorably, and his sight, though injured, was not destroyed, the lens not having been rendered opaque.

It is, however, solely for the purpose of making some practical remarks in reference to operations upon the iris, that I am desirous of drawing attention to the degree of injury this membrane is capable of sustaining, without the occurrence of serious inflammation.

In the extraction of the cataract, the greatest caution is exercised, ordinarily, by operation to avoid wounding the iris, and an insufficient section of the cornea has often been the consequence of extreme carefulness on this point. Mr. Guthrie has endeavoured to mitigate the apprehensions of surgeons with respect to the ill effects of such accidents, and advises that a portion of the iris should be incised by the knife while passing through the anterior chamber, if the tendency of the iris to fold over the knife cannot be prevented but by withdrawing the instrument.

My own experience leads me to similar conclusions; simply incised wounds of the iris, or those where a small portion has been unavoidably cut off, I have never found to lengthen the period of recovery, to give rise to iritis, or to produce subsequent inconvenience.

In the protrusions of the iris which take place after the extraction of the cataract, and keep the divided cornea from uniting, I have found the practice safe and effectual to cut off the prominent portion.

When such protrusions occur in consequence of accidental wounds of the cornea, or when that tunic has been perforated by ulceration, the natural process of cure is, for lymph to be thrown out upon the surface of the exposed iris, and for a new membraneous covering to form, approaching, after a time, to the hardness of cornea; the prolapsed portion gradually diminishes, the spot, where the opening was, becomes opaque, the iris adhering to it and occasioning distortion of the pupil. This process, however, is often very tedious when left to nature, and much inconvenience is experienced by the long continued weakness of the eye. In such cases the prolapsed iris, forming a little bag filled by the aqueous humour behind, acts as a mechanical obstacle to the healing of the cornea, and much suffering is spared the patient, and much progress in the cure effected, by puncturing the protruded iris with a cataract needle, or snipping it off with scissors; in either operation there is an immediate escape of the aqueous humour, the iris falls back within the wound of the cornea, and the hernia seldom recurs. I have never found any inflammation to follow this mode of proceeding.

The liberties that may be taken with the iris is a matter of great importance, with reference to the operations for artificial pupil. I am inclined to believe that many persons with extensive opacities on the cornea, especially in cases where suppurative ophthalmia has pre-existed, are condemned to perpetual blindness, to whom a valuable degree of vision might be given by the removal of the iris opposite any clear portion of cornea that may be left. In every case of the kind, where there is no useful sight, if any of the cornea is clear, and the eye be otherwise healthy, the operation ought to be tried; and I have been surprised to find with how small a portion of cornea remaining transparent, a person may have a degree of vision to some extent useful, and, in a great degree, a source of comfort to him. And in such cases I prefer the operation of tearing away a portion of the iris, and cutting it off. A small section is made in the cornea near to the clear portion, the iris drawn out with a hook or forceps, and as much cut off as is practicable. Care will be necessary in this operation to avoid injuring the crystalline capsule.

These observations refer to wounds of the iris, such as cuts, punctures, and lacerations; contusions of that delicate membrane, and long-continued pressure behind it, as from a displaced lens, or from a portion of the lens, or any more foreign body in the anterior chamber, are usually followed by inflammation of the iris; and in cases of extraction, where the cataract has been forced out through an insufficient corneal section, the iritic attack that too commonly follows, depends probably upon the injurious pressure made upon the iris.

I have remarked a circumstance of a very curious nature in reference to the pupil in cases where this aperture can be of little use if it exist in its customary position, near the centre of the iris. The fact has been presented to me under so many different forms, that I can have no doubt upon the subject, though I have not seen any intimation of the kind in writers on ophthalmic surgery. I am, therefore, desirous of suggesting the point to the attention of those medical men especially who have peculiar facilities of observing diseases of the eyes.

What I refer to is, *the efforts of Nature to form a pupil at that point of the eye where it will be most useful.* In examining eyes where central opacities of the cornea existed, I have been repeatedly struck with the appearance of the pupil, which I have observed to be of a long, narrow shape, as if the longitudinal fibres of the iris had been split for the purpose of producing an aperture exactly opposite the clear portion of cornea, so as to be in the most suitable part for distinct vision. I was inclined to attribute this to some accidental circumstance; and when it existed in persons in whom the opacity of the cornea was occasioned by external injury, I supposed it to depend upon the fortunate coincidence of a wound on that part of the iris at the time when the original injury was inflicted. But having remarked, with much interest, in operations for artificial pupil, that an aperture in the iris, made at some little distance from the clearest portion of cornea, will in time extend itself, so as to be exactly in the position I at first designed and wished it to be, I can have no doubt that occasionally, in other cases of opacity from disease, Nature endeavours to remedy the evil by a similar effort. I am so satisfied of this fact, as regards operations, as to have no hesitation in declaring it, under the hope that others may be able to verify it by their own observations.—*Prov. Med. Journ.*

---

EXTERNAL APPLICATION OF IODINE IN CROUP.

In the 150th number of the Provincial Medical and Surgical Journal, Mr. E. Copeman reports several bad cases of croup in children, in which recovery took place by painting the skin over the larynx and trachea frequently, with a strong tincture of iodine, in conjunction with administration of calomel. The application of the iodine produces, he says, no pain, no inflammation, no vesication (?); and it interferes with no other method of treatment.

---

### THE IMPORTANCE OF ABSTINENCE FROM BREAD IN DIABETIS MELLITUS.

BY THEOPHILUS THOMPSON, M. D.

In this case whenever the use of bread and biscuits was prohibited, and of all vegetables, except the cruciferous order, both the quantity of the urine and its specific gravity were notably decreased. The use of some toasted bread caused the quantity to rise from two to five pints, and the specific gravity increased from 1.027, to 1.041. This happened repeatedly in the course of the case, leaving no doubt of the fact of the influence of a minute portion of bread.—*Provincial Medical and Surgical Journal.* Aug. 5.

---

DR. JAMES ON THE RELIEF OF DEFORMITIES PRODUCED BY CICATRICES IN THE NECK AFTER BURNS.

In a paper read before the Provincial Medical Association, at their recent meeting at Leeds, by Mr. James, he endeavoured to prove the possibility of comple relief in the most aggravated cases of these distressing deformities. In eight cases treated by him the success was complete in seven; in the remaining one the thorough attention which is uniformly necessary, was not paid. Equal success was obtained in by far the greater proportion of cases treated by his colleagues, on his plan.

The principal points of this paper are here briefly recapitulated:—

"That, whereas in the limbs no difficulty exists in maintaining the proper position of the part after the cicatrix has been set free, there being but one joint concerned, and that easily fixed, this is far from being the case in the neck, from the peculiar mobility of that part, arising chiefly from the numerous joints in the cervical spine. It might be supposed, *a priori*, and indeed, has been, that confining the head back would keep the chin and sternum sufficiently asunder; but this is not so. To elude the effect of the cicatrix as it contracts, the cervical spine becomes shortened with a curve either to one side or backwards, as the case may be. To render this approximation impossible became, therefore, the object of my inquiry. I considered that if an apparatus could be interposed between the clavicles and lower jaw, extending backwards to the basis cranii, so as to prevent these parts from approaching each other, I should obviate the difficulty. These purposes are fully attained by the apparatus here presented, which possesses the further advantage of raising the chin by the action of the screw, so that the change in the position, not only of the soft parts, but of the bones, is gradually redressed, and the neck and face wholly, or in a great degree, restored to their former proportions; for it must be observed that the bones themselves, as in talipes or varus, become altered in their shape. The rigid cicatrix holds the chest and front of the face tightly together, so that as the child grows (for it is generally in children these accidents occur, especially females,) the whole bony apparatus is fixed; and when it has been chiefly on one side, I have even seen the orbit of that side considerably lower than the other. I have also seen the lower incisors pushed horizontally by the pressure of the tongue, the counter-pressure of the muscles of the lower lip being wholly wanting.

I do not pretend that all traces of so great a deformity can be effaced by the operation—that there will be no drawing down of the lower lip, no scar, no detriment to the personal appearance, but I am warranted in asserting that the lips will be allowed to close, to retain the saliva, and for distinct articulation—that the head and face may be carried erect, and freely moved, the lower lids no longer everted—that the patient will enjoy her life in comfort, and no more exhibit a picture miserable to behold. It has been objected that the contraction may return. The answer is, that it does not, if the apparatus is worn for a few months after the healing is completed. The observation of many years warrants me in stating this.

I may further and incidentally mention, that the apparatus I have described not only answers the purpose for which it was originally intended, but that, if employed in due time, it is capable of preventing the primary cicatrices consequent on burns from contracting, as is fully shown by a patient, now under the care of my friend and colleague, Mr. Harris, just about to be discharged from this hospital;* *and furthermore, that it may be most advantageously employed in those cases where the cervical vertebræ give way from disease, or where it exists in the processus dentatus, and support is required.*

Such are the uses of the instrument, and such are the advantages of the operation; but it must not be concealed that it is long, sometimes difficult, and very painful—that very great attention is requisite in the subsequent management, and a long confine-

* Eliza James, aged eleven, Exeter Ward. Burn occurred in April, 1842. It was treated without the collar for five months. It then began to contract, and the collar was applied, and continued to the present time. The head is quite upright; the distance from the lower jaw to the clavicle is fully three inches and a half. The mouth not deformed.—June 27, 1843.

ment necessary on the part of the patient; yet, so strong is the feeling in the female mind of the horrid disfigurement, as well as physical disability, produced by these accidents, that I have never met with one patient who has not been deeply grateful for the relief afforded.

In conclusion Mr. James refers to the "valuable memoir" of Professor Mütter, proposing the adoption of the Taliacotian principle, so as to cover the wound formed in the neck after the removal of the cicatrix. He thinks that in very bad cases this will not answer the purpose, unless the collar be also employed. For though separation at the time may be effected between the chin and the sternum, yet without the continued and gradual action of the screw, they will not be restored to their natural position.

I may observe, in this place, that in small cicatrices in any part of the body, I have sometimes adopted the plan of destroying them by caustic potass. The ulcers which form offer no obstacle to any extension which may be wished, and cicatrix which follows has no peculiarity like that consequent on burns by fire, a circumstance sufficiently remarkable in itself.

As the mode of treatment has already been stated in the former paper I have alluded to, I shall not occupy your time by particular details, but merely state that it consists—

1. In dissecting the hardened cicatrix from the subjacent parts, having previously dissected it with a Brodie's knife in most cases, and, in all, forming a flap to turn up under the chin; and I may here take occasion to mention that I think it the safest and best method to operate on the patient in the recumbent posture.

2. In confining this flap under the chin by broad straps of adhesive plaster, and a uniting bandage, secured at the top of the head, which must be shaved to some extent.

3. In covering the large exposed surface in the throat with moistened lint, and bread and water poultices, confined by a paste-board collar, until suppuration is freely established, the head being rather thrown back at the same time.

4. By the use of the screw collar as soon as suppuration is established. In very bad burns it is often desirable to change the first for one with a longer screw, as ground is gained for its action. The apparatus should be worn for many months at least after the cure is completed.

I will not weary your patience by the details of any cases in addition to the three formerly published, to which I beg to refer, but shall merely give a reference to the fourteen I have alluded to, and the names of the surgeons by whom they were performed. Permit me to add, that the fact of the operation being practised by my colleagues to the present time, is, perhaps, the best evidence that can be produced, that it answers the purpose for which it was intended.—*Ibid.*

### PILULA FERRI COMPOSITA.

In order to prepare this pill in such a manner as to keep the carbonate of iron in an undecomposed state, and to insure uniform consistence of the mass, it has been found that the directions given in the Pharmacopœia will be sufficient for these purposes, if the following points be attended to:—Dissolve the sulphate of iron, finely powdered in the treacle, with a moderate heat, and add the carbonate of soda, stirring constantly until the effervescence has entirely ceased, and the mixture has become cool; then add the myrrh gradually, and incorporate the mass. As a little evaporation takes place at the commencement of the process, a small excess of treacle is requisite to supply the deficiency. This mass retains its colour and consistence remarkably well.—*Pharm. Journ.*

### MEETING OF MEDICAL GENTLEMEN IN SUFFOLK.

The first annual meeting of the Suffolk Branch of the Provincial Medical Association was held at the Corn Exchange, Stowmarket, on Friday the 18th inst., and was attended by twenty-three gentlemen of that town, Ipswich, Aspal, Norwich, Mendlesham, Haughley, Bidlestone, Hadleigh, Walsham, Melton, Holbrook, Needham Market, Bury, Sudbury, and Botesdale.

Dr. Durrant, of Ipswich, took the chair, and read a retrospective address on the improvements in, and additions to, the science of medicine made during the last year. The objects of the association, he said, were well known and fully appreciated. It continued to maintain a high reputation and distinguished position—one animus prevading the whole of the members, having equally for its object the advancement of science, the establishment of harmony and good feeling among its associates, and a zealous maintainance of the honor and respectability of the medical profession. The formation of district branches had been productive of very considerable advantages to the association, by keeping alive interest in the general proceedings, stimulating individual members to exertion, and affording opportunities of social and professional intercourse to those whose avocations or other causes did not permit them to attend the more distant meetings. At the last assembly of the Eastern Branch a committee was formed to watch the progress of Medical legislation, in reference to the expected Bill of Sir James Graham. At a very interesting meeting on the subject in Stowmarket, in April last, both Houses of Parliament were petitioned, praying the delay of any legislative measures until the whole subject of medical regulation and reform had been thoroughly investigated. No proceedings, however, up to the present time, had been adopted by Sir James Graham, and from the importance of the parliamentary matter, the entire subject would, he apprehended, undergo further postponement. Among the various contributions to medical science which had been made during the last year, were those of Dr. R. Smith, on Cerebral Disease of Children; Dr. M. Hall on Blood-letting in Apoplexy and Paralysis; Dr. Mayo on Paraplegia; Dr. Debreyne on Epilepsy; Dr. Hennis Green on the Treatment of Cholera by the use of Sulphur-baths, Mr. Miller on Catalepsy; Mr. Jackson on Hydrophobia; Dr. O'Shaughnessy on the use of Indian Hemp; Dr. Weston on the use of Stramonium; Dr. Fontanelle on Hæmoptysis; M. Bondin on Climate in Phthisis; Dr. Bird on Empyema; and Dr. Montgomery on Cancer Uteri. On referring to the obituary, Dr. Durrant added, he found, with pleasure, that the medical profession had to lament fewer deaths during the past than in many of the preceding years. The names of Walker and Tyrrell, of London, would be fresh in the memory of all, coupled with which were those of Barron Larry, Devergie, and Pelletier, of Paris, and Fricke of Hamburg. While others attempted to follow their footsteps, let them faithfully endeavour to ensure the greatest possible amount of benefit to the profession, which, from the large field of observation enjoyed by provincial practitioners, it unquestionably claimed at their hands.

Mr. Bree, of Stowmarket, then read a report relating to medical reform, and a correspondence which he had had, in his official capacity as honorary Secretary, with Sir James Clark, in which he informed Sir James that "a copy of the letter of Sir James on medical reform was directed to be sent to each of the members for the County of Suffolk, intimating that it contained all that they desired on the subject, and requesting them to support any measure embodying its principles." The reply of Sir James contained an expression of his particular pleasure to find "that the subject of medical reform was taken up in so sensible a manner. I look (Sir James added) to the medical practitioners in the provinces as an active means of bringing about that sound reform in the profession which it required for its respectability and for the benefit of the public. I think it very doubtful whether Sir James Graham will bring in his Bill this session, Ministers having so much upon their hands. If so we shall have the summer before us to come to a general agreement upon the heads of such a Bill as will be satisfactory to the great body of medical practitioners. If the profession is true to itself it will get a proper measure of reform."

Cases were then read to the meeting, and recommended to be published in the Society's Transactions. They related to instances of prolapse and inversion of the bladder in a female child; medullary sarcoma of the prostate gland, in a child nine months of age; and ichthyosis simplex and bronchocele successfully treated; and the following subjects were introduced for discussion:—Bleeding in mania; the co-existence of disease of the heart with chorea; opium in strangulated hernia; opium in constipated bowels; paracentesis thoracis in empyema; insectivoria (!) in the intestinal canal.

A discussion was afterwards brought forth on FLUID DEPOSITS IN THE CHEST, AND TAPPING, under such circumstances.

Mr. Martin related a case in which he had tapped the chest several times, and at length left a tube in the aperture for several weeks to conduct off the fluid, with ultimate recovery of the patient.

Dr. Durrant said he had not found ill effects from the admission of air in this operation, and thought its rapid absorption prevented the putrefactive process.

Mr. Bree considered that the air was not absorbed but imbibed, and the difficult physiological point was how the air was removed by fresh chemical combination, absorption. In the practice of Dr. Ranking the fluid in the pleuritic cavity had often been absorbed; he quoted Dr. Hope's cases, and the favorable influence of mercury, and cautioned the profession against the unnecessary performance of tapping in cases where the vital powers, aided by appropriate medicines, would effect a cure by absorption. He had observed bad effects from the introduction of atmospheric air, and that he believed the low fever sometimes following the operation of paracentesis was attributable to the putrefactive process, in consequence of the air entering from without.

Dr. Baird considered it to be still a *quæstio vexata* as to the precise symptoms which should be regarded as demanding tapping.

Mr. Crosse remarked that he should be disinclined to tap for the removal of serous fluid in a patient having any vital powers such as to encourage the hope of absorption; but he should not hesitate to operate where sero-purulent fluid was formed in considerable quantity, creating urgent symptoms, and he should judge of the nature of the fluid by the preceding symptoms and the constitution and temperament of the individual. The great difficulty was in forming an accurate diagnosis; cases of this description afforded the most decided test of the ability of the practitioner. The current of this discussion, in which so many gentlemen spoke from actual experience, marked, he considered, the great progress of medical science. He thought tapping the chest, in a well considered case, not less creditable to the surgeon than lithotomy, or operating for hernia.

Dr. Kirkman spoke of the ill effects of copious bleeding in maniacal cases.

Mr. Crosse, from repeated observation, considered such treatment often led to dementia, or even to permanent idiotcy.

The meeting of next year, it was resolved, should be held at Woodbridge, and Dr. Lynn be requested to preside, and Mr. Bree was requested to act as Secretary for the ensuing year.

The subsequent dinner at the King's Head Inn, was attended also by some non-professional gentlemen of the town, Dr. Durrant in the chair. The usual toasts, and others appropriate to the occasion, were drunk, being the customary marks of notoriety, only distinguished by some nonsence uttered on the subject of medical reform by Mr. Crosse, of Norwich, to the effect that "from medical reform he did not expect much, believing that the profession must look to itself and reform itself, and rather fearing that legislation would be more injurious than otherwise, were no restriction put upon those not legally qualified, to prevent their deceiving and injuring the public as heretofore. Mr. Crosse seems ignorant of the fact, that already the law permits at least one medical body to prosecute unlicensed pretenders to medicine, namely, the worshipful Company of Apothecaries.—*London Lancet*, Aug. 26, 1843.

---

Dr. Robert Lee, in a very talented and learned lecture, in the course of which he cites many authorities to prove the nature of the venous inflammation of the uterus, enumerates a hundred cases from his own practice, in which the patients having died, the ovaria, Fallopian tubes, spermatic veins, &c., were in each case found loaded with pus or lymph, indicating active inflammation of the uterine absorbents and appendages.—*Ibid.*

---

### DR. SIBSON ON THE MEANS OF DETERMINING THE RELATIVE POSITION OF THE THORACIC AND ABDOMINAL VICERA.

At the late anniversary meeting of the Provincial Medical and Surgical Association held at Leeds, Mr. Sibson stated, that in 1836, when examining patients with chest affections, he felt his knowledge of the relative position of the thoracic and abdominal vicera was imperfect and confused. To remedy this he took outline sketches of the viscera from most of the bodies that were dissected in the General Hospital, near Nottingham, at first by the eye, afterwards by the aid of a frame. After some time, Dr. Hodgkin, of London, suggested to Mr. Sibson a very valuable plan, facilitating and rendering more accurate the diagrams. This consisted in placing a frame over which a piece of net or lace was stretched, above the body the organs of which were to be sketched, and by tracing with chalk the outline of each organ, as seen through the gauze. Mr. Sibson then proceeded to give a rapid sketch of the general results obtained by the carrying out of this plan.

The apex of each lung ascends above the clavicle from one to two inches. This is an important practical fact, as by examination of this region tubercles may be detected in their early stage, and long before they have extended to that part of the lung below the clavicle. The inner line of the right lung is behind the sternum for its whole length, whereas that of the centre of left lung diverges obliquely outwards and downwards opposite to the fourth costal cartilage. The lower edge of the right lung is generally behind the sixth costal cartilage; that of the left lung is about a rib's breadth lower. The upper margin of the liver, from the dulness of that organ, forms an admirable means for distinguishing the lower border of the resonant right lung. The hollow resonance of the stomach in contrast with the diffused resonance of the lung enables the lower border of the left lung to be defined with equal facility.

In emphysema the chest is constantly in the state of a very deep inspiration; the lower margin of the lung descends several ribs' breadths below its usual seat; at the same time the diaphragm draws down the lungs; it in a like manner and extent, draws down the heart. The dilatation of the structure of the lungs is due not to frequent coughing, but to constant efforts, by extreme inflation of the lungs, to relieve dyspnœa, the structure of the lungs being in this manner overstretched.

In pneumonia, pleuritis, and general tuberculous infiltration of the whole lung, the affected organ is greatly enlarged, the lower margin descends considerably below its normal position, and the walls of the chest on the diseased side are much more expanded than on the healthy one, but the healthy lung expands more than the diseased one on a deep inspiration. In phthisis affecting the summit of the lung, the mass of the lungs does not appear to be much affected in size. In the consolidation and contraction of lung following pleuritis, the whole structure is diminished and the lower margins are raised, their previous position being occupied by the abdominal viscera.

On artificially distending the pericardial sac, it assumes the form of an oblate spheroid, on the top of which is placed a smaller hemisphere which surrounds the great vessels. In pericarditis with effusion, the sac assumes the same form and displaces the lungs to a considerable extent at the upper part of the sternum; it likewise causes the central tendon of the diaphragm to protrude to a great extent into the abdomen, displacing the liver and stomach. In disease of the heart with enlargement, the lungs are displaced to an extent proportionate to the enlargement, but not at the upper part of the sternum; the liver and stomach, the whole diaphragm, and consequently the basis of both lungs, are considerably lower than in the normal state.

In enlargement of the liver the free play of the lungs and heart is interfered with if the patient lies on the left side, the weight of the liver falling upon the heart interferes with its action, and causes palpitation. In persons affected with ovarian dropsy or ascites, the abdominal viscera, and consequently the diaphragm, are pushed abnormally upwards, and the lower borders of the lungs and heart are unnaturally high.

The same state obtains, but to a more extreme degree, when the stomach or intestines are over distended as in dyspepsia, this state inducing palpitation; or in peritonitis, where the enormous distension often seriously interferes with the action of the heart and lungs. In empyema and its extensive pleuritic effusion the diaphragm is more lowered and the abdominal viscera are more displaced than in any other disease of the lungs.—*Provin. Medical Journal*, Aug. 12, 1843.

---

### RUPTURE OF THE UTERUS—RECOVERY.

The patient in this case was in her nineteenth year, and confined for the first time. Delivery was attempted by the long forceps, but in vain; the head of the infant had to be opened, and delivery was accomplished by means of the hook. In passing the hand into the uterus, a longitudinal rent was discovered, corresponding to the right iliac fossa, and from six to seven centimetres in length. The hand, when passed into this gap in the uterus, came in contact with the mass of the small intestines. A month afterwards the uterus contracted, and the tear in its substance could no longer be perceived. The patient was alarmingly ill. She vomited, had hiccup, violent pain in the abdomen, &c. Nevertheless she did not die; on the contrary, after several days passed in a state between life and death, she began to improve, and finally recovered.—*M. Vaulpre, in Gazette Med. de Paris.*

---

### DR. RICORD ON THE TREATMET OF GONNORRHŒAL OPHTHALMIA.

The diseased parts of the eye must be touched with lunar caustic. The nitrate of silver may be used in solution, in powder, or in a solid pencil. The solution is undoubtedly the easiest of applications. I occasionally use in the following proportions:—

Nitrate of silver, half a drachm:
Distilled water, two drachms.

It is open to this objection, that its action is not limited to the diseased parts, but extends likewise to those which have remained healthy. In infants or refractory adults it is, however, a great resource.

The powder can only be applied in a very unequal manner. I confine its use almost altogether to ulcers of the cornea.

To both, I prefer by far the solid pencil. The inferior lid is first turned down and the pencil carried lightly over it, so as to whiten its surface; for the upper eyelid the same operation is repeated, and such spots of the ocular conjunctiva as happen to be affected must also be touched, but never the cornea.

To protect its transparency oil has been recommended, but this liquid, running over the other parts of the eye, prevents the proper application of the caustic. An injection of water is made immediately after, so as to wash away those portions of nitrate of silver which have remained uncombined. After a first application, should the swelling and pain not be diminished, and the secretions not become thinner, more sanious, and less abundant, a second cauterisation must be made, and this four, five, or six hours after the first. It should be renewed a third, and even a fourth time, at twenty-four hours interval, until diminution of the symptoms is observed.

Œdema of the conjunctiva, producing moderate chemosis, may be left to itself; but if considerable, the late Professor Sanson's advice should be attended to, and the chemosis excised—an operation which should follow, and not precede, cauteristation, in order that the action of the lunar caustic be not interfered with by hæmorrhage.

As to purulent chemosis, I recommend, with Scarpa, free scarification of the phlegmonous swelling.

Although I give unbounded praise to nitrate of silver in all stages of this disease, yet I would not have you believe that I rest upon it exclusively. I derive most powerful assistance from blood-letting, abundant and repeated, both with the lancet and with leeches to the temples, and in the course of the jugular vein, frequent lotions of the eye with a decoction of poppy heads (tepid), neutral salts, as revulsives on the intestinal tube, foot baths, the elevated position of the head, and frictions around the orbit, and in the nares on the affected side, with extract of belladonna, the best sedative in affections of the eye. M. Suhel combines extract of belladonna with an equal quantity of the strong mercurial ointment, and obtains excellent results. Blisters and setons may be advantageously employed after the acute period has gone by. Lastly, I would recommend promptitude and decision in the application of this treatment; it has never failed me but once in the course of many years' practice, and that was the case I have mentioned to you, in which the deceptive mildness of the symptoms was the cause of a fatal hesitation.—*Prov. Med. Journal.*

NEW SYSTEM OF HÆMOSPASY.

In these days of homœopathy and hydropathy, and other humbug devices with names pretending to be Greek, we should regard with suspicion every innovation in medical doctrine or practice which appears in an hellenic garb—a suspicion that is engendered, naturally enough by the probability that alleged discoveries which are announced in dead languages will turn out to be only new contrivances of quacks to gull the inexhaustibly gullible public. There is, however, one new practice, raised under a Greek flag, namely, *hémospasia*, which has for some time deservedly attracted attention in France, and which does not appear to belong to the category of humbugs. Hémospasia forms the subject of an article in the "Gazette Medicale" of the 22d July last, in which journal it has before been noticed, and seems to us to be worthy of serious consideration. It has, moreover, some relation to the *conversazione* of "old friends with new faces," at which we have latterly entertained our readers. We shall here, therefore, introduce it to their notice.

This practice, the name of which is derived form *αιμα*, *blood*, and *σπαω*, *I draw*, or *attract*, consists in effecting revulsion by forming a vacuum over a considerable extent of the surface of the body—of one or two limbs—or even one half of the body. The practice has been introduced by M. Junod, who has invented a particular apparatus adapted for the purpose, and to whom one of the Montyon prizes has been assigned by the Academy of Sciences for this improvement in therapeutics. The Academy has expressed, in two different memoirs, a highly favourable opinion of M. Junod's views and practice, which are alleged to have been tested in the Parisian hospitals with the most satisfactory results.

Having not yet seen the work published by M. Junod, nor the reports of the Academy upon his labours, we are not in a position to enter into particulars with respect to them. We would, however, direct attention to the principle involved in this method of cure, which is evidently the same with that of dry-cupping; namely, that of drawing the fluids *towards* some part of the body, and, consequently, *from* some other part, without diminishing the aggregate quantity of fluid circulating in the whole body at the time. The importance of this intention in many diseases, and chiefly those of a congestive kind, must be sufficiently obvious; yet it seems to have been much better appreciated formerly than at present, since the practice of dry-cupping was very familiar to the ancients, and to the moderns, also, down to the seventeenth century, while, in the present day, it is rarely employed, although we occasionally hear of it.

Considering the *hémospasie* of M. Junod to be what it evidently is, an application of the principle of dry-cupping on an extended scale, it must be regarded merely as a revival of an old practice, although, doubtless, improved in efficacy by the apparatus invented by that gentleman. In the entertaining and instructive work of Prospero Alpini, entitled "De Medicinâ Ægyptiorum," it is remarked that the Egyptian practitioners did not use cupping-glasses to elicit eruptions in malignant fevers, which was a very much esteemed practice among some of the physicians of Europe. "Nam hi (midici nostrates, scil.) in istis febribus applicant ad decem usque et plures, cucurbitulas *universo corpori*, interdum scarificatas, atque interdum *citra scarificationem*, ut ex alto a paribus nobilibus ad cutim venenum avellant."* (Lib. ii., c. 16, *sub initio*, Ed. Venet, 1592.) So that in the sixteenth century it was a frequent practice to use dry-cupping over the whole surface of the body, which must be acknowledged to have been a pretty active *hémospasie*, and the latter practice—so novel that its name has, probably, not yet been heard by the greater part of European practitioners—turns out to be, in some sort, another "old friend with a new face." We mean not to detract, in the smallest degree, from M. Junod's merit in bringing forward the practice, and suggesting the use of apparatus by which it may acquire increased efficiency. We desire merely to illustrate the utility of studying the *old writers on medicine*, which would save ingenious gentlemen of the present day a deal of trouble, and set them about improving what has been already designed, instead of planning what was old even in the time of our grandfathers. Archæology apart, we call the attention of our readers to M. Junod's practice, which seems to be susceptible of some very important applications.—*Lancet*, Aug. 19, 1843.

FORMULA FOR RHEUMATISM.

M. Pereyra, of Bordeaux, who has adopted the use of guaiacum for rheumatic affections in preference to any other vaunted remedy, employs the following formula:—Finely powdered resin of guaiacum, a drachm; orange leaves, powdered, half a drachm; acetate of morphine, three-quarters of a grain. These ingredients are mixed, and divided into sixteen powders, one of which is to be taken every two hours. The acetate of morphine is useful both for enabling the stomach to tolerate the guaiacum and in moderating the stimulant effects of this substance which so often compel its disuse.

*Lond. Lancet.*

SUGAR, according to Dr. Prout, is not unfrequently present in the urine of gouty and dyspeptic persons; and, so far as the experience of that distinguished physician has extended it invariably exists in the urine when the system is affected with carbuncle or malignant boils.—*Dr. Percy.*

* "For these (our own physicians to wit) apply, in those fevers, as many as ten cupping-glasses, or more, sometimes with scarification and sometimes without, in order that they may draw the poison outwards from the noble parts to the skin."

